# The future of CRISPR gene editing according to plant scientists

**DOI:** 10.1016/j.isci.2022.105012

**Published:** 2022-08-25

**Authors:** Job de Lange, Lawton Lanier Nalley, Wei Yang, Aaron Shew, Hans de Steur

**Affiliations:** 1 Department of Agricultural Economics, University of Arkansas, Fayetteville, AR 72701, USA; 2 Department of Agricultural Economics, University of Gent, Gent, Belgium

**Keywords:** Biological sciences, Biotechnology, Plant biology, Plant genetics

## Abstract

This study surveyed 669 plant scientists globally to elicit how (which outcomes of gene editing), where (which continent) and what (which crops) are most likely to benefit from CRISPR research and if there is a consensus about specific barriers to commercial adoption in agriculture. Further, we disaggregated public and private plant scientists to see if there was heterogeneity in their views of the future of CRISPR research. Our findings suggest that maize and soybeans are anticipated to benefit the most from CRISPR technology with fungus and virus resistance the most common vehicle for its implementation. Across the board, plant scientists viewed consumer perception/knowledge gap to be the most impeding barrier of CRISPR adoption. Although CRISPR has been hailed as a technology that can help alleviate food insecurity and improve agricultural sustainability, our study has shown that plant scientists believe there are some large concerns about the consumer perceptions of CRISPR.

## Highlights

• Maize and soybeans are anticipated to benefit the most from CRISPR technology
• Fungus and virus resistance are anticipated to be the vehicle for implementation
• Consumer perception/knowledge gap could impede adoption of CRISPR

## Graphical abstract

**Figure:**
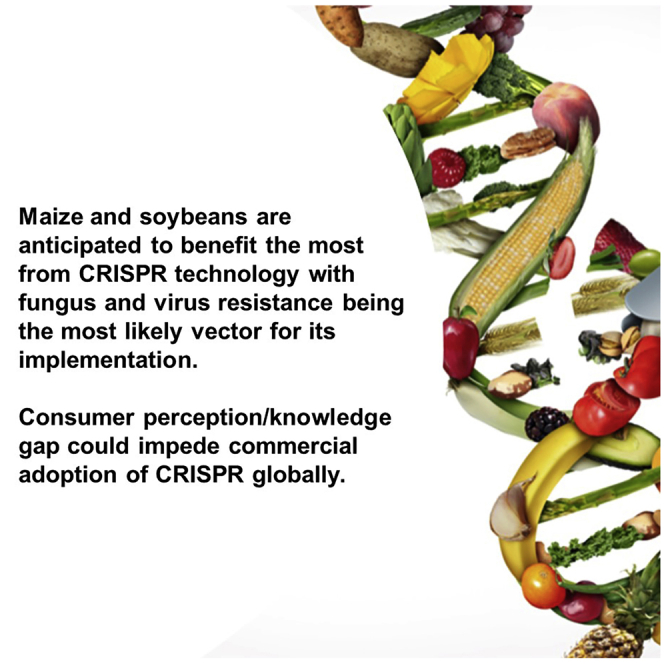

## Introduction

Global food production is under increasing pressure from multiple external factors, including climate change (Hasegawa et al., 2021; Müller et al., 2011; Ray et al., 2019; Rosenzweig et al., 2014), population growth (Charles et al., n.d.; Ray et al., 2013; Tilman et al., 2011; United Nations - Department of Economic and Social Affairs, 2019; van Dijk et al., 2021), and increasing water scarcity (Dolan et al., 2021; Falkenmark, 2013; FAO, 2012). Climate volatility is expected to increase with global climate change, resulting in the emergence and increased incidences of new and existing plant viruses (Chakraborty and Newton, 2011; Chaloner et al., 2021; Karpicka-Ignatowska et al., 2021) and increased pest pressures (Barford, 2013; Bebber et al., 2013; Ma et al., 2021), which have the potential to reduce agricultural productivity and food security (FAO, 2020). The global population is expected to increase to 9.7 billion by 2050 (United Nations - Department of Economic and Social Affairs, 2019), increasing the demand for food globally between 36 and 56% from 2010 output (van Dijk et al., 2021). Historically, plant breeding has been seen as one of the most effective tools for increasing agricultural output and is still viewed as one of the greatest tools for addressing global food insecurity (Qaim, 2016). According to Evenson and Gollin (2003), modern seed varieties contributed almost 40% to the agricultural production growth observed in low-income countries between 1981 and 2000, highlighting the importance of plant science in global food security. However, as yield increases in classical breeding begin to slow there is a need for new breeding techniques to ensure global food security.

New Plant Breeding Techniques (NPBTs) are emerging as a response to both the increasing global food demand and increasing environmental pressure from production agriculture (Enfissi et al., 2021; Qaim, 2020; Schaart et al., 2015; Zaidi et al., 2019; Smith et al., 2021; Van deWiel et al., 2018). Some of these new breeding techniques consist of zinc-finger nuclease, oligonucleotide directed mutagenesis, cisgenesis and intragenesis, RNA-dependent DNA methylation, grafting on GM stock root, reverse breeding, agro-infiltration and synthetic genomics (Lusser et al., 2011).

Gene editing (GEd) technologies, allow plant scientists to alter, delete, and/or add genetic material at site-specific locations in the gene of a living organism. Key differences between GM and GEd are that GEd technologies can make more accurate site-directed insertions in the DNA and that the insertion of foreign DNA from another organism (transgenesis) is less common in gene editing technologies (Ding et al., 2016; Martin-Laffon et al., 2020; Qaim, 2020; Ricroch, 2019). Most GEd applications are INDELs, small insertions or deletions of a few random base pairs in the genome. Examples of existing gene editing technologies are transcription activator-like effector nucleases (TALENs), Zinc-finger nucleases (ZFNs), Meganucleases, Oligonucleotide-Directed Mutagenesis (ODM) and clustered regulatory interspaced short palindromic repeats (CRISPR-Systems). TALENs use engineered nucleases and can create double-stranded breaks (DSBs) everywhere in the genome of a living organism. These breaks are repaired and sequence alterations can be created (Joung and Sander, 2013). ZFNs are a programmable combination of zinc finger domains with an endonuclease which can cut DNA.

CRISPR-Systems act like an adaptive immune system of bacteria and archaea. This immune system has the ability to find and exterminate unwanted DNA in a highly effective and specific manner (Blenke et al., 2016). CRISPR allows scientists to delete certain viruses from plants (Jiang and Doudna, 2017; Manghwar et al., 2019; G. Song et al., 2016). Examples of CRISPR range from improved resistance against rice blast (*Magnaporthe oryzae*) in China (Wang et al., 2016), and the increased shelf-life of tomatoes (Yu et al., 2017). Variations introduced through CRISPR and all GEd techniques might be indistinguishable from conventionally bred counterparts with established analytical tools. The modifications can completely resemble random mutations regardless of being spontaneous or induced chemically or via irradiation. Therefore, if an identification of these organisms is demanded, a new challenge will arise for seed, food, and feed testing laboratories and enforcement institutions (Grohmann et al., 2019).

Although the scientific community has continuously worked to increase the potential and functionality of CRISPR gene editing to contribute to food security and sustainability of agricultural production, the consensus on which crop(s), which trait(s) and which region(s) will benefit the most is still mostly nebulous. Despite its potential, and the large scientific research community both working on and interested in CRISPR the technology faces a litany of barriers to adoption and dissemination (Jorasch, 2020). CRISPR gene editing in food crops, specifically staple non-animal feed crops (rice and wheat), face multiple barriers such as low consumer acceptance, regulatory issues and lack of (technical) infrastructure in different regions. Most existing social science studies on CRISPR gene editing focus on regional case studies and examine the perspectives of consumers and regional plant breeders (Gleim et al., 2019) on the technology, barriers of adoption, possible functions of the gene editing tool and the problems the technology can help to solve. Yet, no study has provided an empirical, global elicitation on the opinions of public and private plant scientists worldwide on the subjects of barriers, functions and benefits for specific crops of CRISPR. This study has gathered scientific opinions across each potential region CRISPR could be deployed, addressing both the private and public sector and over fourteen crops in order to provide an aggregated view on the major drivers, barriers and prospects of CRISPR gene editing. A better understanding of the potential of CRISPR from those on the ground floor of its evolution can help provide a better idea of its future.

### Controversy over CRISPR gene editing applications in agriculture

Existing research has produced several functions of CRISPR technology that could help combat food insecurity through plant breeding: herbicide resistance (Ricroch et al., 2017), drought resistance (Chilcoat et al., 2017), salt soil tolerance (Farhat et al., 2019), insect resistance (Zahoor et al., 2021), biofortification (Chilcoat et al., 2017; Jia and Wang, 2014; Ricroch et al., 2017), fungus resistance (Ricroch et al., 2017), virus resistance (Ali et al., 2016; Wang et al., 2016), increased shelf life (Yu et al., 2017), increased fertilizer use efficiency (Tiwari et al., 2020) all of which have potential to improve the sustainability of agricultural production.

CRISPR has the potential to contribute to the solutions of problems encountered in food production globally, specifically in low-income countries. Feasible beneficiaries of CRISPR gene editing are reduced food insecurity (S. Ahmad et al., 2021; Georges and Ray, 2017; Karavolias et al., 2021; Massel et al., 2021; Y. Zhang et al., 2019; Zhu et al., 2020), reduced environmental damage in agricultural production (S. Ahmad et al., 2021; Biswas et al., 2021; Georges and Ray, 2017; Karavolias et al., 2021; Massel et al., 2021; Y. Zhang et al., 2019; Zhu et al., 2020), increased nutritional value in crops (S. Ahmad et al., 2021; Biswas et al., 2021; Karavolias et al., 2021; Zhu et al., 2020), increased producer profits (S. Ahmad et al., 2021; Van der Oost and Fresco, 2021) and increased yields and reduced yield variability (S. Ahmad et al., 2021; Biswas et al., 2021; Georges and Ray, 2017; Karavolias et al., 2021; Zhu et al., 2020), all of which could play a vital role in increasing regional and global food security via plant breeding.

Despite the perceived benefits of CRISPR, like GM, the technology has also resulted in controversy among consumers, policymakers, and agricultural producers. From the demand side consumer hesitancy to accept GM may be the largest impediment to higher adoption rates among producers. Previous research has found that consumers have low levels of knowledge and numerous misperceptions about GM food. A study in the USA revealed nearly equal numbers of consumers prefer mandatory labeling of foods containing DNA as do those preferring mandatory labeling of GM foods (McFadden and Lusk, 2016). Although consumer acceptance of GM crops is heterogeneous across (Delwaide et al., 2015) and even within countries a meta-analysis found that for specific GM products that consumers were most likely to accept GM oils and least likely to accept GM meat (Lusk et al., 2015). GM products that provided tangible benefits, such as increased nutrition to consumers, significantly decreased premiums for non-GM food. Public opinion polls are often used to characterize consumer sentiment and motivate more precautionary policies for GM food. Whether justifiable or not, consumer concern could lead to a climate that impedes particular research methods and lowers the potential return to investments in biotechnology applications.

Reported risks and barriers of CRISPR gene editing implementation are policy/legal issues around CRSPR gene editing (Andoh, 2017; Menz et al., 2020; Purnhagen, 2018; Smyth et al., 2014), struggling to find competent delivery methods (F. Zhang et al., 2014), lack of fundamental knowledge on gRNA design (Masmitjà et al., 2019; Wilson et al., 2018), intellectual property rights issues (Martin-Laffon et al., 2019; Mulvihill et al., 2017), lack of knowledge and misunderstanding among consumers (Ishii and Araki, 2016; Shew et al., 2018), the risk of off-target effects (N. Ahmad et al., 2020; Graham et al., 2020; X. H. Zhang et al., 2015), the creation of gene drives (Dolezel et al., 2020; Noble et al., 2017), and the high costs of the technology and subsequently underdeveloped infrastructure and technical expertise. These issues may have contributed to the European Court of Justice (ECJ) requiring CRISPR gene edited crops to be subject to traditional GMO regulations in 2018, limiting the applications of the technology and significantly increasing the costs of commercialization of CRISPR gene edited crops (Purnhagen, 2018; Purnhagen and Wesseler, 2020). Alternatively, countries like Argentina and the USA use a case-by-case judgment system to assess whether a CRISPR gene edited organism is GM or not (Menz et al., 2020). Examples are the approved CRISPR/Cas9 edited canola in the USA – regulated like a non-GM crop and under certain scenarios plants containing transgenes or genome edited (multiple or more than one simultaneous modifications such as insertions, deletions, substitutions) are exempt from the USDA biotechnology regulations.

In 2022, China is taking the first regulatory steps to allow gene-edited crops to enter Chinese markets, as it seeks to tackle domestic food insecurity (Patton, 2022). The potential of CRISPR to combat global food insecurity and its controversy among consumers, producers and regulatory bodies before its commercial release highlights the importance of better understanding where (continent) and how (which trait) and what (which crop) CRISPR applications could be implemented in commercial agriculture.

The majority of current social science research on CRIPSR gene editing is either about the benefits, risks and barriers of the technology, or the consumer perceptions of (CRISPR) gene edited foods (Ishii and Araki, 2016; Shew et al., 2018). However, the literature lacks a holistic view on where the CRISPR gene editing sector is moving from a scientific researcher standpoint. Therefore, this study aims to serve as the first step of better understanding what the global plant science community thinks the potential and barriers of CRISPR gene-editing technology are, and where and how CRISPR may emerge in commercial agriculture globally. The results of this study can be used on a granular scale to assess continent-, crop- and sector- (public/private) level issues regarding CRISPR.

## Results

The survey distribution resulted in 1040 unique responses, of which 669 were used in the study. Of the entire sample, 371 responses were not used for comparisons, for one of two reasons. Given the length of the survey and thought which was required, any responses under 2 min were deleted (47 responses). Also, responses with a completion rate (percent of survey questions answered) below 90% were deleted (324 responses). A summary of the profiling variables linked to the 669 participants of this study can be found in Table 1.

**Table 1:** Number of survey respondents, by region and funding sector

	Africa	Asia	Europe	North America	Oceania	South America	Total
Public	124	76	187	162	13	39	601
Private	37	24	105	40	5	21	232
Both	17	13	23	18	3	4	78
Total	178	113	315	220	21	64	911

Note: each participant was able to select that they worked in multiple regions or sectors, therefore the total of 669 respondents resulted in a total of 911 region and sector counts.

### Functions of CRISPR – A regional comparison

Table 2 highlights the mean scores on the potential of successful implementation of possible functions of CRISPR gene editing, separated by region and sector. Respondents rated the functions on a 7-point probability scale (1= low probability, to 7= high probability). The survey did not set out to explain why an issue was important but rather what issue(s)/function(s) plant scientists thought would be successfully addressed via CRISPR.

**Table 2:** Plant scientists’ opinions on the functions of CRISPR gene editing technology, rated on a scale from 1 (low probability) to 7 (high probability)

Functions	Africa		Asia		Europe		North America (ơ= 4,00)^a^		Public		Private	
	(ơ = 3,89)^a^		(ơ = 3,95)^a^		(ơ = 3,56)^a^				(ơ = 3,87)^a^		(ơ = 3,51)^a^	
	Mean	# of responses	Mean	# of responses	Mean	# of responses	Mean	# of responses	Mean	# of responses	Mean	# of responses
Herbicide resistance					3,24-	291	5,02+	196	4,19+	540		
Drought resistance	4,53+	168			3,82-	295			4,13+	560		
Salt soil resistance	2,96-	159			3,04-	291	3,42-	194	3,33-	537	2,63-	215
Insect resistance	4,42+	161			4,01+	290				541	4,00+	215
Biofortification					3,07-	290	3,52-	193	4,21+		2,62-	213
Fungus resistance	4,49+	166	4,60+	98	4,66+	297	4,96+	197	4,71+	548	4,58+	219
Virus resistance	4,85+	164	4,81+	100	4,46+	294	4,74+	196	4,62+	542	4,61+	217
Increased shelf life					3,28-	291						
Fertilizer use efficiency	3,26-	162	3,27-	94	3,18-	288	3,43-	195	3,29-	536	3,01-	214
Improved cultivation	3,45-	159			3,23-	291	3,34-	196	3,33-	536		
Other^b^		62			2,28-	106			2,35-	191	2,38-	93

**Notes:** The presented values denote an issue of the corresponding variable which was statistically (p <0.05) higher (+) or lower (−) than the weighted average of all functions of CRISPR implementation of the corresponding region/sector. An empty/blank cell denotes no statistical difference was found between a specific function and the mean for all functions for a region. No significant differences from the weighted average mean were found for South America and Oceania, therefore these results are not included.

a ơ denotes the weighted average of the aggregated functions of the corresponding region/sector.

b Other consists of answers the respondents were allowed to put forward themselves, examples are: acid soil tolerance, improved seed quality and nitrogen fixation.

African plant scientists rated *drought resistance*, *insect resistance*, *fungus resistance*, and *virus resistance* as the functions of CRISPR gene editing with the highest probability of successful implementation in their region. In Asia, *fungus resistance* and *virus resistance* were the highest rated functions, according to plant scientists active in this region. Plant scientists with research programs focusing on European agriculture, thought *drought resistance*, *insect resistance*, *fungus resistance*, and *virus resistance* were the most likely functions to be successfully implemented. North American plant scientists indicated that *herbicide resistance* will likely be the most successful function, the only function score exceeding five across all regions and sectors.

Four regions (Africa, Asia, Europe, and North America) denoted significantly higher scores for *fungus resistance* and *virus resistance*, possibly because of the expected increase in prevalence of viruses and fungi in agriculture due to climate change. *Drought resistance* and *insect resistance* were reported as potential functions of CRISPR particularly important for Africa and Europe, whereas *herbicide resistance* appears to be dominant in North America according to plant scientists.

### Functions of CRISPR – A public/private comparison

There was more consensus on the potential benefits when comparing private versus public scientists than comparing those from across heterogeneous geographical regions. Public sector scientists rated *herbicide resistance*, *drought resistance, fungus resistance*, and *virus resistance* as the most likely functions to be implemented successfully, regardless of the region they were expected to be implemented in. At private sector level *insect resistance*, *fungus resistance*, *virus resistance*, and *increased shelf life* were perceived as the functions with the highest probability of successful implementation. The public and private sector rated multiple functions of CRISPR similarly. Differences mainly lay in the fact that the public sector scientists viewed *herbicide resistance* and *drought resistance* as feasible functions of CRISPR, whereas the private sector rated these traits not significantly higher.

### Crop benefits of CRISPR implementation – A regional comparison

In Table 3*,* the results on the benefits of CRISPR for specific crops are presented by region and sector. Respondents rated eight crops (respondents could introduce additional crops through the *Other* option) on a 7-point scale of likelihood (1= extremely unlikely, to 7= extremely likely). In all regions except Oceania, *maize* and *soybean* were expected to benefit from CRISPR gene editing. *Wheat* scored significantly higher in all regions, except for Africa and Oceania. Furthermore, *rice* was expected to benefit significantly more compared to the other crops in Asia, North America, and South America. *Potatoes* were expected to benefit in Asia, Europe, and North America, whereas *cassava,* in line with crop production context*,* was only expected to benefit from CRISPR gene editing in Africa.

**Table 3:** Plant scientists’ opinions on the benefits for specific crops of CRISPR gene editing technology, rated on a scale from 1 (extremely unlikely) to 7 (extremely likely)

Crop Benefits	Africa		Asia		Europe		North America (ơ = 3,98)^a^		South America (ơ = 3,80)^a^		Public		Private	
	(ơ = 4,46)^a^		(ơ = 4,21)^a^		(ơ = 3,40)^a^						(ơ = 3,98)^a^		(ơ = 3,66)^a^	
	Mean	# of responses	Mean	# of responses	Mean	# of responses	Mean	# of responses	Mean	# of responses	Mean	# of responses	Mean	# of responses
Wheat			5,16+	86	5,14+	277	5,15+	179	4,76+	46	4,96+	513	5,26+	186
Maize	5,98+	162	5,51+	81	5,13+	275	6,1+	174	5,87+	46	5,61+	508	5,72+	189
Soybean	5,13+	155	5,6+	81	4,5+	268	6,07+	178	6,26+	46	5,2+	505	5,38+	183
Rice			6,33+	88			4,7+	176	4,87+	46	4,71+	504		
Potatoes			4,94+	83	5,12+	274	4,74+	178			4,74+	508	5,02+	191
Cassava	4,97+	159	3,29-	80	1,9-	262	2,5-	173	2,84-	45	3,18-	498	2,27-	183
Sorghum			3,49-	81	2,48-	263			2,69-	45	3,5-	498	2,93-	183
Plantains	3,9-	157	2,43-	79	1,71-	260	2,09-	172	2,32-	44	2,53-	492	1,98-	181
Other^b^	3,66-	593	3,34-	316	2,71-	1032	3,16-	698	2,8-	149	3,24-	1877	2,76-	704

**Notes:** The presented values denote an issue of the corresponding variable which was statistically (p <0.05) higher (+) or lower (−) than the weighted average of all crop benefits of CRISPR implementation of the corresponding region/sector. An empty cell denotes no statistical difference was found. No significant differences from the weighted average mean were found for Oceania; therefore, these results are not included.

a ơ denotes the weighted average of the aggregated crop benefits of the corresponding region/sector.

b Other consists of answers the respondents were allowed to put forward themselves, examples are: *quinoa*, *sugarcane*, *sunflower* and *coffee*.

### Crop benefits of CRISPR implementation – A public/private comparison

Looking at the sectoral level, comparable results were found between plant scientists’ views from the public and private sector. In both sectors, *wheat*, *maize*, *soybean*, and *potatoes* scored significantly higher than other crops. The only difference between the two sectors was the fact that a significantly higher result emerged for *rice* in the public sector, whereas in the private sector no statistical differences were found for *rice*.

### Barriers of CRISPR adoption – A regional comparison

Table 4 shows the perceived barriers of CRISPR gene editing implementation, across different regions and sectors. The survey participants rated nine barriers on a Likert-scale from 1 (strongly disagree) to 7 (strongly agree), which resulted in a mean score for every barrier. Across all regions, *consumer perceptions/knowledge gap* was considered a significant issue impeding the potential adoption of CRISPR. *Policy/legal issues* were also rated significantly higher than the weighted average of the corresponding region in all regions, except South America. *Intellectual property rights issues* were rated as highly impeding in Asia, Europe, and North America. Although the technology is different from GM, the same impediments seem to appear for gene edited crops. Not surprisingly, we see that *high development costs* were considered to be a barrier in Africa and South America. In contrast, off-target effects scored significantly lower in all regions, except Oceania. This could be a function of off-target effects can also occur, and in some cases at a higher rate, in natural mutations and routinely used breeding techniques such as regular crossing or undirected mutagenesis using tissue cultures, chemical mutagens, or irradiation (Modrzejewski et al., 2020). The barrier gRNA design received low scores as well in all regions, except in South America. *Lack of infrastructure/technical expertise* had low scores in the most developed regions in terms of CRIPSR gene editing.

**Table 4:** Plant scientists’ opinions on the barriers of CRISPR gene editing technology, rated on a scale from 1 (strongly disagree) to 7 (strongly agree)

Barriers	Africa		Asia		Europe		North America		Oceania		South America (ơ = 4,10)^a^		Public		Private	
	(ơ = 4,97)^a^		(ơ = 4,24)^a^		(ơ = 4,12)^a^		(ơ = 3,98)^a^		(ơ = 3,98)^a^				(ơ = 4,31)^a^		(ơ = 4,09)^a^	
	Mean	# of responses	Mean	# of responses	Mean	# of responses	Mean	# of responses	Mean	# of responses	Mean	# of responses	Mean	# of responses	Mean	# of responses
Policy/legal issues	5,8+	169	5,45+	99	6,72+	307	4,48+	201	5,22+	18			5,7+	561	5,65+	217
Delivery methods					3,88-	295									3,58-	217
gRNA design	4,59-	169	3,15-	97	2,88-	296	3,29	197	2,5-	18			3,45-	561	3,15-	217
Intellectual property rights			4,8+	94	4,46+	299	4,45+	198					4,57+	561	4,38+	217
Consumer perceptions/	5,46+	167	4,98+	96	5,91+	301	5,29+	198	5,18+	17	5,04+	51	5,51+	561	5,4+	217
knowledge gap																
Off-target effects	3,83-	167	3,75-	96	3,43-	295	3,37-	200			3,14	50	3,56-	561	3,37-	217
Gene drives	3,87-	168			3,37-	299	3,55-	199					3,62-	561	3,46-	217
High development costs	5,67+	166			3,58-	298					4,78+	51			4,36+	217
Lack of infrastructure/	5,71+	170	3,79-	98	2,75-	297	3,3-	199					3,78-	555	3,45-	217
technical expertise																

Note: The presented values denote an issue of the corresponding variable which was statistically (p <0.05) higher (+) or lower (−) than the weighted average of all barriers of CRISPR implementation of the corresponding region/sector. An empty cell denotes no statistical difference was found.

a ơ denotes the weighted average of the aggregated barriers of the corresponding region/sector.

### Barriers of CRISPR adoption – A public/private comparison

The public and private sector plant scientists exhibited similar patterns when it comes to the perception of barriers for CRIPSR gene editing adoption. The key difference is that the private sector considers *high development costs* as a more impeding barrier compared to other barriers where the public sector does not. This could be because CRISPR-Systems come free of charge for public research as long as no commercialization is foreseen.

## Discussion and conclusions

CRISPR gene editing, as one of the main NPBTs, has emerged as a potentially effective tool to address the growing challenges in global food demand and sustainable development. Despite its potential and the growing evidence in research and development, its application to (food) crops has led to a growing controversy, in line to the concerns that feed the public debate on GM technology. This article contributes to the limited research on perceptions of non-consumers by targeting plant scientists. Although the stratified purposeful sample of 669 respondents cannot be considered representative for the plant breeding sector, we have elicited opinions of plant scientists worldwide, covering all regions and including representatives from the private (25%) and public sector (66%), or both (9%). Although all participating plant scientists findings are/could be working with NPBTs, it is important to emphasize that our findings reflect their subjective perceptions on the barriers, functions, and benefits of CRISPR gene edited crops. Whether some barriers will ultimately be a barrier when specific CRISPR applications will be introduced in a region (e.g., consumer acceptance) or whether specific functions will be effectively tackled through CRISPR (e.g., herbicide resistance) is yet to be seen. Nevertheless, our study shows how plant scientists perceive the future of a scientifically promising technology.

Across the board, plant scientists viewed *consumer perception/knowledge gap* to be the most impeding barrier of CRISPR adoption. *Policy/legal issues* were seen to be a significant barrier across all regions, except for South America. This could be explained because of the fact that multiple South American countries have allowed genome edited crops to be grown, such as the production of high oleic soybeans (edited using TALEN gene editing) in Argentina (Menz et al., 2020). Plant scientists working in Europe, denoted the highest score for *policy/legal issues* out of all regions and both sectors. A plausible explanation for this high score of Europe on *policy/legal issues* could be that the European Union has the strictest regulations for CRISPR gene edited crops by making them subject to GM regulations (Purnhagen and Wesseler, 2020). *Intellectual property rights issues* were found to be a significant barrier in Asia, Europe, and North America, the regions which hold the most CRISPR patents in the market (Martin-Laffon et al., 2019). This might be because of the fact that given the large amount of CRISPR patents, there is likely a large amount of copyright infringement or money spent on legal matters protecting that intellectual property. High development costs are seen as a barrier by African and South American scientists; both regions are populated with a high number of developing countries, which likely are plagued by lower relative research and development budgets. In Europe and North America development costs were not perceived as an issue. This could be because of the fact previous research (Bullock et al., 2021) found gene editing required a much smaller potential market area (96% smaller) to break even on the financial investment when compared to genetic modification for the same trait value per hectare.

Overall, across all regions the education of consumers about CRISPR and creating an understandable comprehensive regulatory framework seem to be large impediments of commercial adoption of CRISPR gene editing. In high-income countries, a clear framework for intellectual property rights of CRISPR patents is needed, whereas funding and lack of investment is an impediment in developing countries. In both the public and private sector, *consumer perceptions/knowledge gap*, and *policy/legal issues* seem to be the most impeding barriers of CRISPR adoption, followed by *intellectual property rights issues*. These results are parallel with previous research finding that the largest impediment to GM crops is consumer acceptance. In locations such as the EU, policy and legislative (legal) issues will likely remain an impediment to widescale adoption as law makers or legislative bodies either require mandatory labeling, restrict the cross-border trade of GM products, or simply limit/ban GM production. Given the human and physical capital required to commercialize a GM crop it is not surprising both the private and public sectors worry about intellectual property rights issues in an attempt to either capture value from a commercialized crop or that intellectual property rights may inhibit diffusion of technology. It is important to note that in this survey we cannot ascertain if intellectual property rights are an impeding barrier because of concern of recouping research and development costs or because of the fact that intellectual property rights may slow the transfer of traits across institutions. Further research is needed for this important distinction. In the private sector, scientists see *high development costs* much more as an issue that impedes the adoption of CRISPR adoption.

*Fungus resistance* and *virus resistance* were rated as the most likely functions of CRISPR gene editing to be successfully developed and implemented in agricultural production across four regions (Africa, Asia, Europe, and North America). This should not be surprising given how fungi, viruses, and insects are common issues globally and across most crops. African plant scientists rated *drought resistance* as a likely function to be successfully implemented using CRISPR, not surprising given the decreasing amounts of fresh water available for agricultural production across many parts of Africa. *Insect resistance* was also seen as a likely vehicle for CRISPR in Africa, but also in Europe. *Herbicide resistance* was viewed to be the most likely mode of action in North America, which should not be surprising given the large percentage of adoption of GM herbicide resistant crops available currently across North America. At the sectoral level, both the public and private sectors rated *fungus resistance*, *virus resistance*, and *insect resistance* as the most likely functions to be implemented via CRISPR. Public sector scientists expect *herbicide resistance* and *drought resistance* likely to be implemented as well. The public sector saw biofortification as a function of CRISPR to likely be successful. In the important linkage of supply and demand, previous research has shown consumers were more willing to accept and pay for GM products that provided tangible benefits, such as increased nutrition, compared to GM products in general (Lusk et al., 2015; DeSteur et al., 2015), or even compared to similar non-GM nutritious products (DeSteur et al., 2017).

Our findings suggest that *maize* and *soybean* are expected to benefit the most from CRISPR gene editing across all regions, except for Oceania. *Wheat* (Asia, Europe, North America, and South America), *rice* (Asia, North America, and South America), and *potatoes* (Asia, Europe, and North America) are other crops in which plant scientists globally see potential to benefit from the CRISPR technology. In both the public and private sector, scientists believe that *maize, soybean, wheat*, and *potatoes* are most likely to benefit from CRISPR gene editing technology. The only difference between the two sectors is that public scientists view *rice* as a potential benefactor as well. This may not be surprising given the large role public breeding still plays in *rice* unlike *soy* and *maize.* Either directly related to what plant scientists think will be the crops which will likely benefit the most (maize and soybean) or a spurious correlation, a meta-analysis (Lusk et al., 2015) of consumers found that that GM produced oils (soybean, maize and canola) are most likely to be accepted globally by consumers. Although not specifically tested, given that non-staple crops used for oils fiber, and animal feeds are the most accepted forms of GM crops by consumers may have influenced the plant scientists interviewed in this survey about which crops could benefit the most from CRISPR.

Although CRISPR has been hailed as a technology that can help alleviate food insecurity and improve agricultural sustainability, our study has shown that there are some large concerns among plant breeders about the consumer perceptions of CRISPR gene edited food, policies and legal frameworks concerning the technology, intellectual property rights, and high development costs in low-income countries. Thus, for CRISPR to achieve its full potential, consumers need to be made aware of its benefits both to producers and the environment, and policy makers need a better understanding of why CRISPR is needed (virus/fungus/herbicide/drought resistance). Although CRISPR is not a silver bullet for food insecurity or sustainability, plant scientists fear its true potential may never be realized if consumers and policymakers are not convinced or unaware of its benefits. As gene editing research continues to evolve, more and more tools like CRISPR will be available to plant scientists, but without more economic, legal, and policy support, the adoption of such technologies will continue to lag. A better understanding of how these tools could work, where they could work, and what impediments will keep them from working are vitally important in trying to feed a growing planet with less resources, increasing population, and a more variable climate. Although more research is needed to further examine perceptions and expectations of both scientific and non-scientific stakeholders regarding CRISPR, attempts to provide insights into how plant scientists across regions and sectors see this promising technology evolving in real life. As such, it illustrates the value of socio-economic research on CRISPR technology, alongside public and private efforts in R&D.

### Research methodology and sampling

In this study, we implemented an online survey targeting plant scientists globally. Our survey was directed at a broad scope of plant scientists (plant biology, plant pathology, plant physiology, entomology, and/or plant breeding) who are/could be working with NPBTs. Although the targeted plant scientists were heterogeneous in their disciplines (ranging from private to public institutions, working in different regions and on many different crops), they all had fundamental technical knowledge of plants and could potentially reflect on the challenges and opportunities for a technology such as CRISPR.

The research participants were targeted through stratified purposeful sampling and contact details of plant scientists were derived by conducting an online search of websites of plant scientist platforms, societies, universities, and private companies worldwide. Contact details of scientists who published work related to or focusing on CRISPR gene editing technology were also obtained from the Web of Knowledge database, resulting in the collection of contact details for almost 3,500 plant scientists. The final database consisted of 6,294 unique e-mail addresses of plant scientists, to whom our survey was distributed.

The survey asked each participant their activity in the plant science sector (public/private or both), which regions their research activities primarily focus on (Africa, Asia, Europe, North America, Oceania, South America), and whether their research group/department is active in CRISPR research and development, and if so, for how many years. Each participant was able to select that they worked in multiple regions so the total number of responses could be higher than the total number of participants as a participant who responded they worked in both Africa on maize and Asia on rice were treated as two separate responses. Respondents could only select one primary funding vehicle though, public, private or both. This assumption was needed to delineate the two funding sources, but future research should consider a sliding scale of what percentage of funding is from public and private sources as they often bleed into each other.

Participants were asked to rate the probability of successful development and implementation of different functions of CRIPSR in agriculture on a Likert scale from 1 (low probability) to 7 (high probability), with an ‘I do not know’ option. The potential CRISPR attributes that participants could choose from were herbicide resistance (Ricroch et al., 2017), drought resistance (Chilcoat et al., 2017), salt soil tolerance (Farhat et al., 2019), insect resistance (Zahoor et al., 2021), biofortification (Chilcoat et al., 2017; Jia and Wang, 2014; Ricroch et al., 2017), fungus resistance (Ricroch et al., 2017), virus resistance (Ali et al., 2016; Wang et al., 2016), increased shelf-life (Yu et al., 2017), fertilizer use efficiency (Tiwari et al., 2020), and improved cultivation of crops. Because these benefits are not exhaustive, respondents were allowed to add additional functions in the ‘Other’ box.

Respondents were then asked to rate the barriers to CRISPR adoption, on a Likert scale from 1 (strongly disagree) to 7 (strongly agree). The barriers to widespread adoption choices were policy/legal issues around CRSPR gene editing (Andoh, 2017; Menz et al., 2020; Purnhagen, 2018; Smyth et al., 2014), struggling to find competent delivery methods (F. Zhang et al., 2014), lack of fundamental knowledge on gRNA design (Masmitjà et al., 2019; Wilson et al., 2018), intellectual property rights issues (Martin-Laffon et al., 2019; Mulvihill et al., 2017), lack of knowledge and misunderstanding among consumers (Ishii and Araki, 2016; Shew et al., 2018), the risk of off-target effects (N. Ahmad et al., 2020; Graham et al., 2020; X. H. Zhang et al., 2015), the creation of gene drives (Dolezel et al., 2020; Noble et al., 2017), and the high costs of the technology and subsequent lack of infrastructure and technical expertise.

Finally, respondents were asked to rate the crops which would benefit the most in their opinion from CRISPR gene editing in their region of work (Africa, Asia, Europe, North America, Oceania, South America), on a scale from 1 (extremely unlikely) to 7 (extremely likely). The respondents were also provided with an ‘*I do not know’* option. The list of food crop choices provided in the survey was based on the production data of food crops globally from the Food and Agricultural Organization (FAO, 2019), which were: *wheat, maize, soybean, rice, potatoes, cassava, sorghum, millet, yams, plantains, vegetables, fruits, legumes*, *and other*. For *vegetables, fruits, legumes* and *other* there was a text box available, in which the respondent was asked to specify which crop. The survey in its entirety can be located in Data S1.

All questions on the barriers, functions, and benefits for specific crops were separated by both region and sector (public/private), to allow testing for differences between geographic areas and the public and private sector. University of Arkansas Internal review board (IRB) approval was granted for this survey on 03/08/2020 (protocol number 2102314838). Participants were provided with a written consent form which they had to agree to before the beginning of the survey which stated they could quit the survey at any time. Further, the consent form informed participants their responses would be anonymous with no way to identify individual participants from their respective responses. The survey was initially sent out on 04/22/2021 and a reminder e-mail was administered one month later with the survey closing on 06/22/2021.

### Data analysis and statistical testing

After collecting the responses, statistical analyses were performed on the different variables of the survey questions. All questions were answered on a scale from one to seven and consequently a mean score could be derived from every variable in the survey, separated by region and sector. All descriptive statistics were extracted from Qualtrics and compared. Pairwise t-tests were used for further analysis. The statistical analysis focused on three questions: functions of CRISPR, barriers of CRISPR implementation, and crop benefits of CRISPR application.

For each variable, a weighted average mean was calculated from all scores given by the respondents, per region and sector. Each variable score was compared to the weighted average mean within its region or sector, using a pairwise t-test. In this way, outlying scores could be detected, scores which significantly differ from the weighted average of all scores. For example, results can be compared for barriers of CRISPR adoption in Africa or the beneficiaries of CRISPR in North America. Using this method, we assessed whether the respondents rated certain functions, barriers, and crop benefits significantly higher or lower relative to others, separated by region and sector.

A significance level of 5% was used for all tests. The results of significance levels use a multiple comparison (for example, if we simultaneously test that drought resistance and insect resistance are significantly higher than the average of all traits in Africa) correction. The p-values estimated were adjusted pvalues using Benjamini–Hochberg (BH) method. Pairwise t-tests were only run within a region or sector, as comparing between regions and/or sectors is difficult because of major context differences. However, this research draws a picture of where the major difficulties, opportunities, and benefits of CRISPR gene editing lay by region and sector.

### Limitations of the study

A major limitation of this study was the fact we forced participants to choose between public, private or both, when selecting from their primary funding agencies for CRISPR research. Future research should allow for a percentage from each to get a more precise picture of where CRISPR funding is being sourced. Furthermore, allowing participants to choose multiple crops instead of one primary prevented the comparison of funding differences between crops as the survey did not ask participants to disaggregate their funding (if they choose “both”) between multiple crops. Future research may want to ask participants to select the primary crop they work on. We had a disproportionate number of public responses as the public sector provides more readily available contact information. Future research may want to target the private sector more strategically.

## STAR★Methods

### Key resources table

**Table:** 

REAGENT or RESOURCE	SOURCE	IDENTIFIER
**Deposited data**		

Raw and analyzed data	This paper	Data S1 and S2

**Software and algorithms**		

Excel	Microsoft	https://www.microsoft.com/en-us/microsoft-365/excel

### Resource availability

#### Lead contact

Further information and requests should be directed to and will be fulfilled by the lead contact, Lawton Lanier Nalley (llnalley@uark.edu).

#### Materials availability

This study did not generate new unique reagents.

### Experimental model and subject details

All participants were over the age of 18. Sex and gender data was not collected on participants as it was not a factor analyzed in the study. University of Arkansas Internal review board (IRB) approval was granted for this survey on 03/08/2020 (protocol number 2102314838). Informed consent was given by all participants.

### Quantification and statistical analysis

A significance level of five percent was used for all tests. The results of significance levels use a multiple comparison correction. The p-values estimated were adjusted p values using Benjamini–Hochberg (BH) method. Pairwise t-tests were only run within a region or sector, as comparing between regions and/or sectors is difficult due to major context differences.

## Data Availability

• Data used in this study is available in the supplemental file entitled Data S2: Survey Results • This paper does not report original code. Any additional information required to reanalyze the data reported in this paper is available from the lead contact upon request.

## Supplemental information

Data S1. Complete survey used to estimate Tables 1–4

Data S2. Survey results is related to the supplemental file
