# Supplementary material for: The future of CRISPR gene editing according to plant scientists
Source: iScience. 2022 Aug 25;25(9):105012. doi: 10.1016/j.isci.2022.105012 (PMC9460836; doi:10.1016/j.isci.2022.105012)
Supplement: Data S1. Complete survey used to estimate Tables 1–4 [file mmc1.pdf]

iScience, Volume *25*

Supplemental information

The future of CRISPR gene editing according to plant scientists

Job de Lange, Lawton Lanier Nalley, Wei Yang, Aaron Shew, and Hans de Steur

Data S1: Complete Survey used to Estimate Tables 1 – 4.

CRISPR Drivers, Barriers & Prospects - A comparative study among US, EU and African plant scientists

---

Start of Block: Introduction block

Q32 Thank you for your participation!
In this study we are interested in your perceptions about the drivers, barriers and prospects of the CRISPR gene editing technology, with a focus on the technology´s potential for the production of food crops. Your opinion is important to us and we hope that you will take the time to give us your insights.

Risks and Benefits: Your participation will assist in the advancement of CRISPR gene editing technology and give insights in the drivers, barriers and prospects among plant scientists in Europe, Africa and North America concerning the technology. There are no anticipated risks to participating in this study.

There is no compensation for your time, which we estimate will take approximately **5 minutes**.

Voluntary Participation: Your participation in the research is completely voluntary.

Confidentiality: Your responses on the survey will be recorded anonymously. Only basic demographic information (i.e. age, gender, education etc.) will be collected.

Right to Withdraw: You are free to refuse to participate in the research and to stop participation during the survey if you choose.

If you have questions or concerns about this study, you may contact llnalley@uark.edu. For questions or concerns about your rights as a research participant, please contact Ro Windwalker, the University's Compliance Coordinator, at 1+ (479) 575-2208 or by e-mail at irb@uark.edu.

Q31 I am over the age of 18 and I would like to participate in this research

○ Yes (1)

○ No (2)

Skip To: End of Survey If Q31 = No

Q58 By continuing and completing this survey, I am agreeing for my anonymous responses to be used in this research.

○ Continue (1)

○ Do not continue (2)

Skip To: End of Survey If Q58 = Do not continue

Page Break

Q41 Please indicate your academic level:

- ○ High school (4)
- ○ Bachelor of Science (1)
- ○ Master of Science (2)
- ○ Doctor of Philosophy (Ph.D). (7)
- ○ Postdoctoral Researcher (3)
- ○ Professorship (6)
- ○ Other (If yes, please specify) (5) ____

Q27 Are you active in the public or private plant science sector?

- ○ Public (1)
- ○ Private (2)
- ○ Both (please specify in the next question) (3)
- ○ I am not active in the plant science sector (4)

Skip To: End of Survey If Q27 = I am not active in the plant science sector

---

Display This Question:

If Q27 = Both (please specify in the next question)

Q1 Please indicate how much of your time (in %) you are active in the public and/or private plant science sector:

	Public sector					%			Private sector		
	0	10	20	30	40	50	60	70	80	90	100
Distribution public/private sector ()											

Page Break

Q2 How many years of experience do you have in the plant science sector?

- 0-10 years (4)
- 10-20 years (5)
- 20-30 years (6)
- 30-40 years (7)
- 40+ years (8)

Q56 Please indicate how much of your time (in %) you are active in the fundamental and/or applied plant sciences:

	Fundamental plant sciences	%	Applied plant sciences
	0 10 20 30 40 50 60 70 80 90 100		
Distribution fundamental/applied plant sciences ()			

Q55
The focus in my role as plant scientist is mainly on (please specify using the sliders, in %, adding to a total of 100%):

	%	%
	0 10 20 30 40 50 60 70 80 90 100	
Yield improvement ()		
Stress resilience (biotic/abiotic) ()		
Nutritional alteration/improvement ()		
Mineral uptake (e.g. fertilizer efficiency) ()		
Storage (e.g. shelf life) ()		
Other (please specify) ()		

Page Break

Q25 Which crops are you primarily working in?

- ☐ Wheat (1)
- ☐ Maize (2)
- ☐ Soybean (3)
- ☐ Rice (4)
- ☐ Potatoes (5)
- ☐ Cassava (7)
- ☐ Sorghum (8)
- ☐ Millet (9)
- ☐ Yams (10)
- ☐ Plantains (11)
- ☐ Vegetables (if yes, please specify) (15) ____
- ☐ Fruits (if yes, please specify) (16) ____
- ☐ Legumes (if yes, please specify) (17) ____
- ☐ Other (if yes, please specify) (6) ____

Q4 Which regions are of primary focus concerning the R&D activities of your research group/department?
*Note: please indicate in which countries the **department you work in** is actively conducting R&D*

- ☐ Africa (1)
- ☐ Asia (2)
- ☐ Europe (3)
- ☐ Oceania (5)
- ☐ North America (4)
- ☐ South America (6)

Page Break

End of Block: Interoduction block

Start of Block: CRISPR gene editing activity & budgets

Q13 Is your research group/department active in CRISPR gene editing Research & Development?

- Yes (1)
- No (2)

Display This Question:

If Q13 = Yes

Q5 When did your research group/department start their CRISPR gene editing Research & Development?
Note: Starting in this context means, R&D budget was allocated for the first time to explore the potential of the CRISPR gene editing technology.

- Before 2013 (13)
- 2013 (4)
- 2014 (5)
- 2015 (6)
- 2016 (7)
- 2017 (8)
- 2018 (9)
- 2019 (10)
- 2020 (11)
- 2021 (12)

Display This Question:

If Q13 = Yes

Q6 Could you indicate for your research group/department, what percentage of the total R&D budget is **currently** allocated to CRISPR gene editing Research & Development?

% % Not Applicable

0 10 20 30 40 50 60 70 80 90 100

% of R&D budget currently allocated to CRISPR gene editing ()	

Q7 Could you indicate for your research group/department, what percentage of the total R&D budget do you envision **will be** allocated to CRISPR gene editing Research & Development in 3, 5 and 10 years?

	%		%		Not Applicable					
	0	10	20	30	40	50	60	70	80	90	100
in 3 years ()											
in 5 years ()											
in 10 years ()											

Page Break

End of Block: CRISPR gene editing activity & budgets

Start of Block: Applications & Barriers

Display This Question:

If Q4 = Africa

Q23 Given your research activities, how do you rate the probability of successful development and implementation of the following possible functions of the CRISPR gene editing technology in **Africa**? Rate each on a scale from 1 to 7.

Note: succesful development and implementation in this context means that the corresponding function can succesfully be developed for, and applied to multiple crops grown in the region.

	1 (Low probability) (85)	2 (86)	3 (87)	4 (Medium probability) (88)	5 (89)	6 (90)	7 (High probability) (91)	8 I don´t know (92)
Herbicide resistance (1)	o	o	o	o	o	o	o	o
Drought resistance (2)	o	o	o	o	o	o	o	o
Salt soil resistance (3)	o	o	o	o	o	o	o	o
Insect resistance (4)	o	o	o	o	o	o	o	o
Biofortification (5)	o	o	o	o	o	o	o	o
Fungus resistance (6)	o	o	o	o	o	o	o	o
Viruses resistance (7)	o	o	o	o	o	o	o	o
Increased shelf life (9)	o	o	o	o	o	o	o	o
Fertilizer use efficiency (10)	o	o	o	o	o	o	o	o
Improved cultivation (11)	o	o	o	o	o	o	o	o
Other (if yes, please specify) (8)	o	o	o	o	o	o	o	o

Display This Question:

If Q4 = Asia

Q39 Given your research activities, how do you rate the probability of successful development and implementation of the following possible functions of the CRISPR gene editing technology in **Asia**? Rate each on a scale from 1 to 7.

Note: succesful development and implementation in this context means that the corresponding function can succesfully be developed for and applied to multiple crops grown in the region.

	1 (Low probability) (23)	2 (24)	3 (25)	4 (Medium probability) (26)	5 (27)	6 (28)	7 (High probability) (29)	8 I don´t know (30)
Herbicide resistance (1)	○	○	○	○	○	○	○	○
Drought resistance (2)	○	○	○	○	○	○	○	○
Salt soil resistance (3)	○	○	○	○	○	○	○	○
Insect resistance (4)	○	○	○	○	○	○	○	○
Biofortification (5)	○	○	○	○	○	○	○	○
Fungus resistance (6)	○	○	○	○	○	○	○	○
Viruses resistance (7)	○	○	○	○	○	○	○	○
Increased shelf life (9)	○	○	○	○	○	○	○	○
Fertilizer use efficiency (10)	○	○	○	○	○	○	○	○
Improved cultivation (11)	○	○	○	○	○	○	○	○
Other (if yes, please specify) (8)	○	○	○	○	○	○	○	○

Display This Question:

If Q4 = Europe

Q38 Given your research activities, how do you rate the probability of successful development and implementation of the following possible functions of the CRISPR gene editing technology in **Europe**? Rate each on a scale from 1 to 7.

Note: succesful development and implementation in this context means that the corresponding function can succesfully be developed for and applied to multiple crops grown in the region.

	1 (Low probability) (30)	2 (31)	3 (32)	4 (Medium probability) (33)	5 (34)	6 (35)	7 (High probability) (36)	8 I don´t know (37)
Herbicide resistance (1)	o	o	o	o	o	o	o	o
Drought resistance (2)	o	o	o	o	o	o	o	o
Salt soil resistance (3)	o	o	o	o	o	o	o	o
Insect resistance (4)	o	o	o	o	o	o	o	o
Biofortification (5)	o	o	o	o	o	o	o	o
Fungus resistance (6)	o	o	o	o	o	o	o	o
Viruses resistance (7)	o	o	o	o	o	o	o	o
Increased shelf life (9)	o	o	o	o	o	o	o	o
Fertilizer use efficiency (10)	o	o	o	o	o	o	o	o
Improved cultivation (11)	o	o	o	o	o	o	o	o
Other (if yes, please specify) (8)	o	o	o	o	o	o	o	o

Display This Question:

If Q4 = Oceania

Q37 Given your research activities, how do you rate the probability of successful development and implementation of the following possible functions of the CRISPR gene editing technology in **Oceania**? Rate each on a scale from 1 to 7.

Note: succesful development and implementation in this context means that the corresponding function can succesfully be developed for and applied to multiple crops grown in the region.

	1 (Low probability) (23)	2 (24)	3 (25)	4 (Medium probability) (26)	5 (27)	6 (28)	7 (High probability) (29)	8 I don´t know (30)
Herbicide resistance (1)	o	o	o	o	o	o	o	o
Drought resistance (2)	o	o	o	o	o	o	o	o
Salt soil resistance (3)	o	o	o	o	o	o	o	o
Insect resistance (4)	o	o	o	o	o	o	o	o
Biofortification (5)	o	o	o	o	o	o	o	o
Fungus resistance (6)	o	o	o	o	o	o	o	o
Viruses resistance (7)	o	o	o	o	o	o	o	o
Increased shelf life (9)	o	o	o	o	o	o	o	o
Fertilizer use efficiency (10)	o	o	o	o	o	o	o	o
Improved cultivation (11)	o	o	o	o	o	o	o	o
Other (if yes, please specify) (8)	o	o	o	o	o	o	o	o

Display This Question:

If Q4 = North America

Q36 Given your research activities, how do you rate the probability of successful development and implementation of the following possible functions of the CRISPR gene editing technology in **North America**? Rate each on a scale from 1 to 7.

Note: succesful development and implementation in this context means that the corresponding function can succesfully be developed for and applied to multiple crops grown in the region.

	1 (Low probability) (23)	2 (24)	3 (25)	4 (Medium probability) (26)	5 (27)	6 (28)	7 (High probability) (29)	8 I don´t know (30)
Herbicide resistance (1)	◯	◯	◯	◯	◯	◯	◯	◯
Drought resistance (2)	◯	◯	◯	◯	◯	◯	◯	◯
Salt soil resistance (3)	◯	◯	◯	◯	◯	◯	◯	◯
Insect resistance (4)	◯	◯	◯	◯	◯	◯	◯	◯
Biofortification (5)	◯	◯	◯	◯	◯	◯	◯	◯
Fungus resistance (6)	◯	◯	◯	◯	◯	◯	◯	◯
Viruses resistance (7)	◯	◯	◯	◯	◯	◯	◯	◯
Increased shelf life (9)	◯	◯	◯	◯	◯	◯	◯	◯
Fertilizer use efficiency (10)	◯	◯	◯	◯	◯	◯	◯	◯
Improved cultivation (11)	◯	◯	◯	◯	◯	◯	◯	◯
Other (if yes, please specify) (8)	◯	◯	◯	◯	◯	◯	◯	◯

Display This Question:

If Q4 = South America

Q35 Given your research activities, how do you rate the probability of successful development and implementation of the following possible functions of the CRISPR gene editing technology in **South America**? Rate each on a scale from 1 to 7.

Note: succesful development and implementation in this context means that the corresponding function can succesfully be developed for and applied to multiple crops grown in the region.

	1 (Low probability) (37)	2 (38)	3 (39)	4 (Medium probability) (40)	5 (41)	6 (42)	7 (High probability) (43)	8 I don´t know (44)
Herbicide resistance (1)	○	○	○	○	○	○	○	○
Drought resistance (2)	○	○	○	○	○	○	○	○
Salt soil resistance (3)	○	○	○	○	○	○	○	○
Insect resistance (4)	○	○	○	○	○	○	○	○
Biofortification (5)	○	○	○	○	○	○	○	○
Fungus resistance (6)	○	○	○	○	○	○	○	○
Viruses resistance (7)	○	○	○	○	○	○	○	○
Increased shelf life (9)	○	○	○	○	○	○	○	○
Fertilizer use efficiency (10)	○	○	○	○	○	○	○	○
Improved cultivation (11)	○	○	○	○	○	○	○	○
Other (if yes, please specify) (8)	○	○	○	○	○	○	○	○

Page Break

Display This Question:

If Q4 = Africa

Q14 Given your research activities, please give your opinion about what the major barriers are that impede the large scale implementation of CRISPR gene editing in **<u>Africa:</u>**

	Strongly agree (49)	Agree (50)	Somewhat agree (51)	Neither agree nor disagree (52)	Somewhat disagree (53)	Disagree (54)	Strongly disagree (55)
**Policy/Legal issues** are a major barrier for CRISPR gene editing implementation in Africa (1)	○	○	○	○	○	○	○
Struggling to find competent **delivery methods** are a major barrier for CRISPR gene editing implementation in Africa (2)	○	○	○	○	○	○	○
Lack of fundamental knowledge about **gRNA design** is a major barrier for CRISPR gene editing implementation in Africa (3)	○	○	○	○	○	○	○
**Intellectual property rights issues** are a major barrier for CRISPR gene editing implementation in Africa (4)	○	○	○	○	○	○	○
**Consumer perceptions and lack of knowledge on CRISPR gene edited foods** are a major barrier for CRISPR gene editing implementation in Africa (5)	○	○	○	○	○	○	○
**Off-target effects** are a major barrier for CRISPR gene editing implementation in Africa (6)	○	○	○	○	○	○	○
**The risk of spreading of genetic adaptation into the environment (Gene Drives)** is a major barrier for CRISPR gene editing implementation in Africa (7)	○	○	○	○	○	○	○

**High development and implementation costs** are a major barrier for CRISPR gene editing implementation in Africa (10)	○	○	○	○	○	○	○
**The lack of sufficient infrastructure and technical expertise** are a major barrier for CRISPR gene editing implementation in Africa (11)	○	○	○	○	○	○	○

Display This Question:

If Q4 = Asia

Q43 Given your research activities, please give your opinion about what the major barriers are that impede the large scale implementation of CRISPR gene editing in **Asia:**

	Strongly agree (13)	Agree (14)	Somewhat agree (15)	Neither agree nor disagree (16)	Somewhat disagree (17)	Disagree (18)	Strongly disagree (19)
**Policy/Legal issues** are a major barrier for CRISPR gene editing implementation in Asia (1)	○	○	○	○	○	○	○
Struggling to find competent **delivery methods** are a major barrier for CRISPR gene editing implementation in Asia (2)	○	○	○	○	○	○	○
Lack of fundamental knowledge about **gRNA design** is a major barrier for CRISPR gene editing implementation in Asia (3)	○	○	○	○	○	○	○
**Intellectual property rights issues** are a major barrier for CRISPR gene editing implementation in Asia (4)	○	○	○	○	○	○	○
**Consumer perceptions and lack of knowledge on CRISPR gene gene edited foods** are a major barrier for CRISPR gene editing implementation in Asia (5)	○	○	○	○	○	○	○
**Off-target effects** are a major barrier for CRISPR gene editing implementation in Asia (6)	○	○	○	○	○	○	○
**The risk of spreading of genetic adaptation into the environment (Gene Drives)** is a major barrier for CRISPR gene editing implementation in Asia (7)	○	○	○	○	○	○	○

**High development and implementation costs** are a major barrier for CRISPR gene editing implementation in Asia (10)	○	○	○	○	○	○	○
**The lack of sufficient infrastructure and technical expertise** are a major barrier for CRISPR gene editing implementation in Asia (11)	○	○	○	○	○	○	○

Display This Question:

If Q4 = Europe

Q44 Given your research activities, please give your opinion about what the major barriers are that impede the large scale implementation of CRISPR gene editing in **Europe:**

	Strongly agree (13)	Agree (14)	Somewhat agree (15)	Neither agree nor disagree (16)	Somewhat disagree (17)	Disagree (18)	Strongly disagree (19)
**Policy/Legal issues** are a major barrier for CRISPR gene editing implementation in Europe (1)	○	○	○	○	○	○	○
Struggling to find competent **delivery methods** are a major barrier for CRISPR gene editing implementation in Europe (2)	○	○	○	○	○	○	○
Lack of fundamental knowledge about **gRNA design** is a major barrier for CRISPR gene editing implementation in Europe (3)	○	○	○	○	○	○	○
**Intellectual property rights issues** are a major barrier for CRISPR gene editing implementation in Europe (4)	○	○	○	○	○	○	○
**Consumer perceptions and lack of knowledge on CRISPR gene edited foods** are a major barrier for CRISPR gene editing implementation in Europe (5)	○	○	○	○	○	○	○
**Off-target effects** are a major barrier for CRISPR gene editing implementation in Europe (6)	○	○	○	○	○	○	○
**The risk of spreading of genetic adaptation into the environment (Gene Drives)** is a major barrier for CRISPR gene editing implementation in Europe (7)	○	○	○	○	○	○	○

**High development and implementation costs** are a major barrier for CRISPR gene editing implementation in Europe (10)	○	○	○	○	○	○	○
**The lack of sufficient infrastructure and technical expertise** are a major barrier for CRISPR gene editing implementation in Europe (11)	○	○	○	○	○	○	○

Display This Question:

If Q4 = Oceania

Q45 Given your research activities, please give your opinion about what the major barriers are that impede the large scale implementation of CRISPR gene editing in **<u>Oceania:</u>**

	Strongly agree (13)	Agree (14)	Somewhat agree (15)	Neither agree nor disagree (16)	Somewhat disagree (17)	Disagree (18)	Strongly disagree (19)
**Policy/Legal issues** are a major barrier for CRISPR gene editing implementation in Oceania (1)	o	o	o	o	o	o	o
Struggling to find competent **delivery methods** are a major barrier for CRISPR gene editing implementation in Oceania (2)	o	o	o	o	o	o	o
Lack of fundamental knowledge about **gRNA design** is a major barrier for CRISPR gene editing implementation in Oceania (3)	o	o	o	o	o	o	o
**Intellectual property rights issues** are a major barrier for CRISPR gene editing implementation in Oceania (4)	o	o	o	o	o	o	o
**Consumer perceptions and lack of knowledge on CRISPR gene edited foods** are a major barrier for CRISPR gene editing implementation in Oceania (5)	o	o	o	o	o	o	o
**Off-target effects** are a major barrier for CRISPR gene editing implementation in Oceania (6)	o	o	o	o	o	o	o
**The risk of spreading of genetic adaptation into the environment (Gene Drives)** is a major barrier for CRISPR gene editing implementation in Oceania (7)	o	o	o	o	o	o	o

**High development and implementation costs** are a major barrier for CRISPR gene editing implementation in Oceania (10)	○	○	○	○	○	○	○
**The lack of sufficient infrastructure and technical expertise** are a major barrier for CRISPR gene editing implementation in Oceania (11)	○	○	○	○	○	○	○

Display This Question:

If Q4 = North America

Q46 Given your research activities, please give your opinion about what the major barriers are that impede the large scale implementation of CRISPR gene editing in **North America:**

	Strongly agree (13)	Agree (14)	Somewhat agree (15)	Neither agree nor disagree (16)	Somewhat disagree (17)	Disagree (18)	Strongly disagree (19)
**Policy/Legal issues** are a major barrier for CRISPR gene editing implementation in North America (1)	○	○	○	○	○	○	○
Struggling to find competent **delivery methods** are a major barrier for CRISPR gene editing implementation in North America (2)	○	○	○	○	○	○	○
Lack of fundamental knowledge about **gRNA design** is a major barrier for CRISPR gene editing implementation in North America (3)	○	○	○	○	○	○	○
**Intellectual property rights issues** are a major barrier for CRISPR gene editing implementation in North America (4)	○	○	○	○	○	○	○
**Consumer perceptions and lack of knowledge on CRISPR gene edited foods** are a major barrier for CRISPR gene editing implementation in North America (5)	○	○	○	○	○	○	○
**Off-target effects** are a major barrier for CRISPR gene editing implementation in North America (6)	○	○	○	○	○	○	○
**The risk of spreading of genetic adaptation into the environment (Gene Drives)** is a major barrier for CRISPR gene editing implementation in North America (7)	○	○	○	○	○	○	○

**High development and implementation costs** are a major barrier for CRISPR gene editing implementation in North America (10)	○	○	○	○	○	○	○
**The lack of sufficient infrastructure and technical expertise** are a major barrier for CRISPR gene editing implementation in North America (11)	○	○	○	○	○	○	○

Display This Question:

If Q4 = South America

Q47 Given your research activities, please give your opinion about what the major barriers are that impede the large scale implementation of CRISPR gene editing in **South America:**

	Strongly agree (13)	Agree (14)	Somewhat agree (15)	Neither agree nor disagree (16)	Somewhat disagree (17)	Disagree (18)	Strongly disagree (19)
**Policy/Legal issues** are a major barrier for CRISPR gene editing implementation in South America (1)	o	o	o	o	o	o	o
Struggling to find competent **delivery methods** are a major barrier for CRISPR gene editing implementation in South America (2)	o	o	o	o	o	o	o
Lack of fundamental knowledge about **gRNA design** is a major barrier for CRISPR gene editing implementation in South America (3)	o	o	o	o	o	o	o
**Intellectual property rights issues** are a major barrier for CRISPR gene editing implementation in South America (4)	o	o	o	o	o	o	o
**Consumer perceptions and lack of knowledge on CRISPR gene edited foods** are a major barrier for CRISPR gene editing implementation in South America (5)	o	o	o	o	o	o	o
**Off-target effects** are a major barrier for CRISPR gene editing implementation in South America (6)	o	o	o	o	o	o	o
**The risk of spreading of genetic adaptation into the environment (Gene Drives)** is a major barrier for CRISPR gene editing implementation in South America (7)	o	o	o	o	o	o	o

**High development and implementation costs** are a major barrier for CRISPR gene editing implementation in South America (10)	○	○	○	○	○	○	○
**The lack of sufficient infrastructure and technical expertise** are a major barrier for CRISPR gene editing implementation in South America (13)	○	○	○	○	○	○	○

Page Break

Display This Question:

If Q4 = Africa

Q15 What is in your opinion the likelihood of the following crops to benefit significantly from the CRISPR gene editing technology in **Africa**? Rate each on a scale from 1 to 7.

	Extremely likely (71)	Moderately likely (72)	Slightly likely (73)	Neither likely nor unlikely (74)	Slightly unlikely (75)	Moderately unlikely (76)	Extremely unlikely (77)	I don´t know (78)
Wheat (1)	○	○	○	○	○	○	○	○
Maize (2)	○	○	○	○	○	○	○	○
Soybean (3)	○	○	○	○	○	○	○	○
Rice (4)	○	○	○	○	○	○	○	○
Potatoes (5)	○	○	○	○	○	○	○	○
Cassava (8)	○	○	○	○	○	○	○	○
Sorghum (9)	○	○	○	○	○	○	○	○
Millet (10)	○	○	○	○	○	○	○	○
Yams (11)	○	○	○	○	○	○	○	○
Plantains (12)	○	○	○	○	○	○	○	○
Vegetables (if yes, please specify) (17)	○	○	○	○	○	○	○	○
Fruits (if yes, please specify) (18)	○	○	○	○	○	○	○	○
Legumes (if yes, please specify) (19)	○	○	○	○	○	○	○	○
Other (if yes, please specify) (16)	○	○	○	○	○	○	○	○

Display This Question:

If Q4 = Asia

Q48 What is in your opinion the likelihood of the following crops to benefit significantly from the CRISPR gene editing technology in **Asia**? Rate each on a scale from 1 to 7.

	Extremely likely (71)	Moderately likely (72)	Slightly likely (73)	Neither likely nor unlikely (74)	Slightly unlikely (75)	Moderately unlikely (76)	Extremely unlikely (77)	I don´t know (78)
Wheat (1)	○	○	○	○	○	○	○	○
Maize (2)	○	○	○	○	○	○	○	○
Soybean (3)	○	○	○	○	○	○	○	○
Rice (4)	○	○	○	○	○	○	○	○
Potatoes (5)	○	○	○	○	○	○	○	○
Cassava (8)	○	○	○	○	○	○	○	○
Sorghum (9)	○	○	○	○	○	○	○	○
Millet (10)	○	○	○	○	○	○	○	○
Yams (11)	○	○	○	○	○	○	○	○
Plantains (12)	○	○	○	○	○	○	○	○
Vegetables (if yes, please specify) (13)	○	○	○	○	○	○	○	○
Fruits (if yes, please specify) (14)	○	○	○	○	○	○	○	○
Legumes (if yes, please specify) (15)	○	○	○	○	○	○	○	○
Other (if yes, please specify) (6)	○	○	○	○	○	○	○	○

Display This Question:

If Q4 = Europe

Q49 What is in your opinion the likelihood of the following crops to benefit significantly from the CRISPR gene editing technology in **Europe**? Rate each on a scale from 1 to 7.

	Extremely likely (71)	Moderately likely (72)	Slightly likely (73)	Neither likely nor unlikely (74)	Slightly unlikely (75)	Moderately unlikely (76)	Extremely unlikely (77)	I don´t know (78)
Wheat (1)	○	○	○	○	○	○	○	○
Maize (2)	○	○	○	○	○	○	○	○
Soybean (3)	○	○	○	○	○	○	○	○
Rice (4)	○	○	○	○	○	○	○	○
Potatoes (5)	○	○	○	○	○	○	○	○
Cassava (8)	○	○	○	○	○	○	○	○
Sorghum (9)	○	○	○	○	○	○	○	○
Millet (10)	○	○	○	○	○	○	○	○
Yams (11)	○	○	○	○	○	○	○	○
Plantains (12)	○	○	○	○	○	○	○	○
Vegetables (if yes, please specify) (13)	○	○	○	○	○	○	○	○
Fruits (if yes, please specify) (14)	○	○	○	○	○	○	○	○
Legumes (if yes, please specify) (15)	○	○	○	○	○	○	○	○
Other (if yes, please specify) (6)	○	○	○	○	○	○	○	○

Display This Question:

If Q4 = Oceania

Q50 What is in your opinion the likelihood of the following crops to benefit significantly from the CRISPR gene editing technology in **Oceania**? Rate each on a scale from 1 to 7.

	Extremely likely (71)	Moderately likely (72)	Slightly likely (73)	Neither likely nor unlikely (74)	Slightly unlikely (75)	Moderately unlikely (76)	Extremely unlikely (77)	I don´t know (78)
Wheat (1)								
Maize (2)								
Soybean (3)								
Rice (4)								
Potatoes (5)								
Cassava (8)								
Sorghum (9)								
Millet (10)								
Yams (11)								
Plantains (12)								
Vegetables (if yes, please specify) (13)								
Fruits (if yes, please specify) (14)								
Legumes (if yes, please specify) (15)								
Other (if yes, please specify) (6)								

Display This Question:

If Q4 = North America

Q51 What is in your opinion the likelihood of the following crops to benefit significantly from the CRISPR gene editing technology in **North America**? Rate each on a scale from 1 to 7.

	Extremely likely (71)	Moderately likely (72)	Slightly likely (73)	Neither likely nor unlikely (74)	Slightly unlikely (75)	Moderately unlikely (76)	Extremely unlikely (77)	I don´t know (78)
Wheat (1)								
Maize (2)								
Soybean (3)								
Rice (4)								
Potatoes (5)								
Cassava (8)								
Sorghum (9)								
Millet (10)								
Yams (11)								
Plantains (12)								
Vegetables (if yes, please specify) (13)								
Fruits (if yes, please specify) (14)								
Legumes (if yes, please specify) (15)								
Other (if yes, please specify) (6)								

Display This Question:

If Q4 = South America

Q52 What is in your opinion the likelihood of the following crops to benefit significantly from the CRISPR gene editing technology in **South America**? Rate each on a scale from 1 to 7.

	Extremely likely (71)	Moderately likely (72)	Slightly likely (73)	Neither likely nor unlikely (74)	Slightly unlikely (75)	Moderately unlikely (76)	Extremely unlikely (77)	I don´t know (78)
Wheat (1)	○	○	○	○	○	○	○	○
Maize (2)	○	○	○	○	○	○	○	○
Soybean (3)	○	○	○	○	○	○	○	○
Rice (4)	○	○	○	○	○	○	○	○
Potatoes (5)	○	○	○	○	○	○	○	○
Cassava (8)	○	○	○	○	○	○	○	○
Sorghum (9)	○	○	○	○	○	○	○	○
Millet (10)	○	○	○	○	○	○	○	○
Yams (11)	○	○	○	○	○	○	○	○
Plantains (12)	○	○	○	○	○	○	○	○
Vegetables (if yes, please specify) (13)	○	○	○	○	○	○	○	○
Fruits (if yes, please specify) (14)	○	○	○	○	○	○	○	○
Legumes (if yes, please specify) (15)	○	○	○	○	○	○	○	○
Other (if yes, please specify) (6)	○	○	○	○	○	○	○	○

Page Break

Display This Question:

If Q4 = Africa

Q17 What are (or will be) the major beneficiaries of CRISPR gene editing adoption in **Africa**? Rate each option on a scale from 1 to 7.

	1 (No beneficiary) (1)	2 (4)	3 (5)	4 (Medium beneficiary) (2)	5 (6)	6 (7)	7 (Major beneficiary) (3)
Reduced food insecurity (1)	○	○	○	○	○	○	○
Reduced environmental damage in agricultural production (2)	○	○	○	○	○	○	○
Increased nutritional value in crops (3)	○	○	○	○	○	○	○
Increased producer profits (4)	○	○	○	○	○	○	○
Increased yields (5)	○	○	○	○	○	○	○
Reduced yield variability (6)	○	○	○	○	○	○	○
Other (if yes, please specify) (8)	○	○	○	○	○	○	○

Display This Question:

If Q4 = Asia

Q53 What are (or will be) the major beneficiaries of CRISPR gene editing adoption in **Asia**? Rate each option on a scale from 1 to 7.

	1 (No beneficiary) (1)	2 (4)	3 (5)	4 (Medium beneficiary) (2)	5 (6)	6 (7)	7 (Major beneficiary) (3)
Reduced food insecurity (1)	○	○	○	○	○	○	○
Reduced environmental damage in agricultural production (2)	○	○	○	○	○	○	○
Increased nutritional value in crops (3)	○	○	○	○	○	○	○
Increased producer profits (4)	○	○	○	○	○	○	○
Increased yields (5)	○	○	○	○	○	○	○
Reduced yield variability (6)	○	○	○	○	○	○	○
Other (if yes, please specify) (8)	○	○	○	○	○	○	○

Display This Question:

If Q4 = Europe

Q52 What are (or will be) the major beneficiaries of CRISPR gene editing adoption in **Europe**? Rate each option on a scale from 1 to 7.

	1 (No beneficiary) (1)	2 (4)	3 (5)	4 (Medium beneficiary) (2)	5 (6)	6 (7)	7 (Major beneficiary) (3)
Reduced food insecurity (1)	◯	◯	◯	◯	◯	◯	◯
Reduced environmental damage in agricultural production (2)	◯	◯	◯	◯	◯	◯	◯
Increased nutritional value in crops (3)	◯	◯	◯	◯	◯	◯	◯
Increased producer profits (4)	◯	◯	◯	◯	◯	◯	◯
Increased yields (5)	◯	◯	◯	◯	◯	◯	◯
Reduced yield variability (6)	◯	◯	◯	◯	◯	◯	◯
Other (if yes, please specify) (8)	◯	◯	◯	◯	◯	◯	◯

Display This Question:

If Q4 = Oceania

Q51 What are (or will be) the major beneficiaries of CRISPR gene editing adoption in **Oceania**? Rate each option on a scale from 1 to 7.

	1 (No beneficiary) (1)	2 (4)	3 (5)	4 (Medium beneficiary) (2)	5 (6)	6 (7)	7 (Major beneficiary) (3)
Reduced food insecurity (1)	○	○	○	○	○	○	○
Reduced environmental damage in agricultural production (2)	○	○	○	○	○	○	○
Increased nutritional value in crops (3)	○	○	○	○	○	○	○
Increased producer profits (4)	○	○	○	○	○	○	○
Increased yields (5)	○	○	○	○	○	○	○
Reduced yield variability (6)	○	○	○	○	○	○	○
Other (if yes, please specify) (8)	○	○	○	○	○	○	○

Display This Question:

If Q4 = North America

Q50 What are (or will be) the major beneficiaries of CRISPR gene editing adoption in **North America**? Rate each option on a scale from 1 to 7.

	1 (No beneficiary) (1)	2 (4)	3 (5)	4 (Medium beneficiary) (2)	5 (6)	6 (7)	7 (Major beneficiary) (3)
Reduced food insecurity (1)	○	○	○	○	○	○	○
Reduced environmental damage in agricultural production (2)	○	○	○	○	○	○	○
Increased nutritional value in crops (3)	○	○	○	○	○	○	○
Increased producer profits (4)	○	○	○	○	○	○	○
Increased yields (5)	○	○	○	○	○	○	○
Reduced yield variability (6)	○	○	○	○	○	○	○
Other (if yes, please specify) (8)	○	○	○	○	○	○	○

Display This Question:

If Q4 = South America

Q49 What are (or will be) the major beneficiaries of CRISPR gene editing adoption in **South America**? Rate each option on a scale from 1 to 7.

	1 (No beneficiary) (1)	2 (4)	3 (5)	4 (Medium beneficiary) (2)	5 (6)	6 (7)	7 (Major beneficiary) (3)
Reduced food insecurity (1)	◯	◯	◯	◯	◯	◯	◯
Reduced environmental damage in agricultural production (2)	◯	◯	◯	◯	◯	◯	◯
Increased nutritional value in crops (3)	◯	◯	◯	◯	◯	◯	◯
Increased producer profits (4)	◯	◯	◯	◯	◯	◯	◯
Increased yields (5)	◯	◯	◯	◯	◯	◯	◯
Reduced yield variability (6)	◯	◯	◯	◯	◯	◯	◯
Other (if yes, please specify) (8)	◯	◯	◯	◯	◯	◯	◯

Page Break

End of Block: Applications & Barriers

Start of Block: Statements & Final remarks

Q53 To what extent do you agree with the following statements on CRISPR gene editing:

	Strongly agree (1)	Agree (2)	Somewhat agree (3)	Neither agree nor disagree (4)	Somewhat disagree (5)	Disagree (6)	Strongly disagree (7)
CRISPR gene edited foods should be subject to Genetically Modified Organisms regulation. (1)	o	o	o	o	o	o	o
CRISPR gene editing can be one of the major contributors to the solutions of environmental and food insecurity issues (3)	o	o	o	o	o	o	o
CRISPR gene editing technology is currently too expensive to make it a feasible option for developing countries (4)	o	o	o	o	o	o	o
Off-targeted editing is a significant threat for CRISPR gene editing in plant breeding (5)	o	o	o	o	o	o	o
Potential negative side-effects of CRISPR gene editing, have not yet been investigated thoroughly enough to bring gene edited food crops to the market (8)	o	o	o	o	o	o	o
CRISPR gene editing patents will primarily be owned by large plant breeding multinationals (7)	o	o	o	o	o	o	o

In 25 years, the majority of food crops grown globally will be edited using CRISPR gene editing technology (9)	○	○	○	○	○	○	○
The private sector will dominate the CRISPR gene editing market in terms of patents and edited crops on the market, rather than the public sector (10)	○	○	○	○	○	○	○
The CRISPR gene editing market will be dominated by multinationals, startups and scaleups will play a minor role (11)	○	○	○	○	○	○	○
CRISPR gene editing will remain an expensive technology and therefore primarily be applied in developed countries (12)	○	○	○	○	○	○	○

Page Break

Q21 If you have any final remarks concerning the answers you gave in the survey or the questions that were asked, please leave them here.
Also, in case you are interested in the results of this study please leave your e-mail address here for future correspondence.

____

____

____

____

____

End of Block: Statements & Final remarks
